# Health and Diseases of Koalas

**DOI:** 10.3390/ani12081005

**Published:** 2022-04-13

**Authors:** Natasha Speight

**Affiliations:** School of Animal and Veterinary Sciences, Faculty of Sciences, Roseworthy Campus, University of Adelaide, Adelaide, SA 5371, Australia; natasha.speight@adelaide.edu.au

## 1. Introduction

The koala (*Phascolarctos cinereus*) is an arboreal marsupial that is found throughout much of eastern and southeastern Australia, and it relies primarily on eucalypt trees for food, water and shelter. Due to this specialisation, koalas are particularly vulnerable to the effects of habitat loss and fragmentation, which have contributed to the decline of many populations.

This Special Issue of *Animals* brings together a diversity of research on koala health and disease that contributes to our understanding of the various threats to koalas; describes findings that improve the care, monitoring and welfare of individuals; and reviews strategies to safeguard populations. With the recent announcement of the endangered status of many populations, koala health research will be increasingly vital to the support of conservation efforts.

## 2. Health and Diseases of Koalas in This Special Issue

Ongoing koala population declines have been exacerbated by the recent 2019–2020 Black Summer bushfires, which occurred in prime koala habitats in multiple regions across Australia. In South Australia, a massive bushfire burnt half of Kangaroo Island, and whilst hundreds of koalas were rescued as part of an emergency response [1], many perished; this reduced one of the few *Chlamydia*-free koala populations in Australia [2] to only 20% of its pre-fire size.

*Chlamydia* infection and disease is common in other captive and wild koala populations across eastern Australia, and due to this, it is necessary for ongoing research to advance preventative strategies such as vaccination [3]. The successful treatment of individual koalas with chlamydial disease is also important, and it has recently been recognised that many antibiotics [4]—as well as analgesics used for pain relief [5]—can have altered pharmacokinetics in koalas, which may change their efficacy. Hence, this pharmacological research is key to guiding veterinary interventions for rehabilitating koalas.

Recent research using advanced molecular techniques has shown that the characterisation of *Chlamydia* and other infections of koalas, particularly koala retrovirus (KoRV), is complex. KoRV infection exhibits differences within and between populations; these include endogenous and exogenous KoRV subtypes, which can be associated with variable immune responses [6]. Hence, we need to continue to improve our understanding of KoRV epidemiology and pathogenicity, including the differences that occur with KoRV infections in southern koalas [7]. This will allow the prognosis for KoRV-infected individuals to be better predicted, and preventative approaches, such as vaccination [8], to be considered as a strategy to protect koalas in the future.

The likelihood and outcomes of *Chlamydia* and KoRV-associated disease in koalas may also be affected by stress. By using the faecal pellets of captive and wild koalas, researchers have determined cortisol measures in relation to the assessment of metabolite decay [9], biological variation [10], and the effects of captivity [11]. These studies will improve our understanding of cortisol measurement in koalas, and thus, our interpretation of stress and its interactions with their health status.

## 3. Conclusions

Monitoring koala health at individual and population levels clearly needs to be prioritised to maximise the survival chances of this unique species into the future. Each of the many facets of koala research contributes to this goal, and the publications in this Special Issue have significantly improved our increasing knowledge base of the biology, health and diseases of this iconic Australian marsupial, the koala.

## Data Availability

Not applicable.
